# A Distributed Geo-Routing Algorithm for Wireless Sensor Networks

**DOI:** 10.3390/s90604083

**Published:** 2009-05-27

**Authors:** Gyanendra Prasad Joshi, Sung Won Kim

**Affiliations:** Department of Information and Communication Engineering, Yeungnam University, Gyeongsang buk-do 712-749, Korea; E-Mail: joshi@ynu.ac.kr

**Keywords:** geo-routing, communication void, greedy routing, wireless sensor networks

## Abstract

Geographic wireless sensor networks use position information for greedy routing. Greedy routing works well in dense networks, whereas in sparse networks it may fail and require a recovery algorithm. Recovery algorithms help the packet to get out of the communication void. However, these algorithms are generally costly for resource constrained position-based wireless sensor networks (WSNs). In this paper, we propose a void avoidance algorithm (VAA), a novel idea based on upgrading virtual distance. VAA allows wireless sensor nodes to remove all stuck nodes by transforming the routing graph and forwarding packets using only greedy routing. In VAA, the stuck node upgrades distance unless it finds a next hop node that is closer to the destination than it is. VAA guarantees packet delivery if there is a topologically valid path. Further, it is completely distributed, immediately responds to node failure or topology changes and does not require planarization of the network. NS-2 is used to evaluate the performance and correctness of VAA and we compare its performance to other protocols. Simulations show our proposed algorithm consumes less energy, has an efficient path and substantially less control overheads.

## 1. Introduction

Geographic routing, also called position-based routing, geo-routing, location-based routing, or directional routing, was originally proposed for packet radio networks in the 1980s [1-2]. Geographic routing exploits position information instead of topological connectivity information to move data packets to gradually approach and eventually reach the intended destination. Geographic routing eliminates some of the limitations of topology-based routing by using the physical position of the participating nodes as additional information. Each node determines its own position through low energy consumable and low cost GPS or similar positioning services [3-5]

Geographic routing does not require the establishment or maintenance of routes. The localized operation and the state-less feature of geographic routing make it simple and scalable. A new paradigm of geographic routing called geocasting [6], which supports delivery of packets to all the nodes in a given geographic region, made this field more interesting.

Geographic routing for WSNs has been attracting research interest. Most of the existing geographic routing protocols use greedy routing to forward packets from source to destination. Greedy routing is a simple, efficient and scalable strategy for geographic WSNs. Since greedy routing makes pure local decisions, it requires only a simple beaconing protocol. Thus, it consumes considerably less bandwidth than protocols that distribute state globally throughout the network. It is robust under topological changes, because a node can make correct forwarding decisions without requiring up-to-date state of nodes beyond a single hop. Due to their low processing and memory cost, greedy routing is efficient in resource constrained WSNs. In greedy routing, a source node selects a neighboring node that is closest (with respect to Euclidian distance) to the destination as the next hop, until the destination is reached. Similarly, each intermediate node selects a next hop node closest to the destination until the packet reaches the destination. The position of the packet destination is carried in the header of the packet so that intermediate nodes can learn the packet's destination.

**Figure 1.**
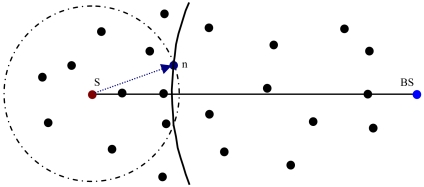
Greedy forwarding.

In Figure 1, the arrow shows the radius of the radio range of sensor S. Whenever S has a packet for the base station (BS), it forwards the packet to n, as its next hop node, because the distance between n and BS is less than any other of neighbor of S to BS. This greedy forwarding process repeats until the packet reaches BS.

**Figure 2.**
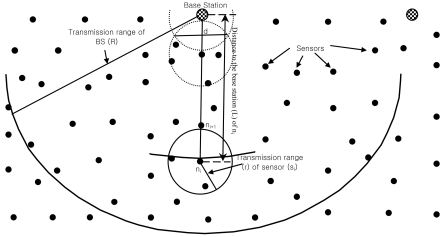
Forwarding area of a sensor in sensor networks, stuck node and void.

Greedy forwarding performs well in dense networks, whereas in sparse networks it does not perform well due to communication voids. A communication void is a state where all neighbor nodes are further away from the destination than the node holding the current packet. The node where the packet gets stuck is called a stuck node, or void node. The packet tarries at the stuck node that has no neighbor closer to the destination than itself. In this situation, packets have to be discarded by the stuck node, when only a greedy forwarding strategy is used, even though a topologically valid path to the destination node may still exist. A communication void is often called a routing hole, dead end or void. In Figure 2, node n_i_ is a node holding the current packet and BS is the destination. Node n_i_ does not have a node, within its forwarding area (a), closer to the BS than n_i_ itself. Let R be the maximum transmission range of BS and L_i_ be the distance to the BS from node n_i_. Assuming that all the sensors are homogeneous with the transmission range r, the forwarding area for node n_i_ depends on the distance to the BS (L_i_) from node n_i_. When L is extremely larger then r, the forwarding area (a_i_) ≈ ½πr^2^. On the other hand, if the BS is very close to the node i.e. L≈r, then the forwarding area (a_Δi_) is smaller than a_i_. i.e. a_Δi_ < a_i_ and can be written as the area of the asymmetric lens $r^{2}=\cos^{-1}\left(\frac{d}{r}\right)-d\sqrt{r^{2}-d^{2}}<\frac{1}{2}\pi r^{2}$, where, d is a triangular height. The forwarding area for any node in the network is in between a_Δi_ to a_i_. A node is a stuck node if there is no node in its forwarding area which is based on L_i_. Node n_i_ is a stuck node in Figure 2. We discuss more about stuck nodes in later sections.

The packet should be forwarded to the closest backward node, if no nodes are available in the forward direction to counter this problem. This may cause looping, circumvented when packets are forwarded only toward the destination with positive progress (i.e. reducing distance).

Although a dense deployment of sensor nodes can reduce the chances a void in the network, it is still possible for some packets to encounter voids, induced by obstacles, unreliable sensors, weak sensor batteries, sensors destroyed due to natural calamities or disasters, etc. For example, sensors that are deployed to monitor wild habitats could be destroyed if a forest fire broke out. Thus, to acquire data from the remaining sensors, until the defunct sensors are replaced, it is imperative to design a void handling technique for geographic routing in an effective and efficient manner.

The communication void problem in greedy forwarding is an important issue in geographical routing. Many protocols have been proposed in the literature to avoid communication voids. Most existing position-based routing protocols have two modes: (a) greedy forwarding mode and (b) recovery mode. If a sender cannot locate a next-hop node that has positive progress toward the packet destination, it switches to recovery mode and attempts to route the packet around the void. Current solutions to this problem are inadequate, as they memorize the path or they cannot find considerably shorter paths.

The key contribution of this paper is to design a communication void-free geographic routing for WSNs. Several routing algorithms exist in the literature [7-18,20,21,29-31]. Most of the existing solutions that switch to recovery mode after encountering the communication void have too much routing overheads and consume more energy. After implementing our algorithm, the network becomes communication void-free if there exists a valid route to the destination. It is not necessary to enter a void handling mode. The proposed method does not recover from a dead end, but restricts entry to the dead end.

The remainder of this paper is organized as follows. Section 2 overviews prior related literature. In Section 3, we describe our proposed algorithm. In Section 4, we compare the performance of our proposed algorithm to other methods. In Section 5, we summarize the work in this paper and describe possible future research that builds upon this study.

## 2. Related work

Routing protocols for position-based wireless sensor networks are presented in the literature. One-hop flooding [7], partial hop-by-hop routing in Geographic Routing Algorithm (GRA) [8], and Partial Source Routing (PSR) in On-demand Geographic Forwarding (OGF) [9] protocols are based on flooding techniques. These flooding-based geographic routing algorithms exploit the simplest communications techniques in a network, i.e. flooding, to locate a stuck data packet and get around a void. Most of these protocols guarantee packet delivery for connected graphs [7].

Some of these protocols execute full flooding, a technique to send a stuck packet to all network nodes. Flooding is inefficient in terms of resource utilization. Some efficient full flooding algorithms [10] and some restricted flooding mechanisms have been proposed in the literature to minimize the occurrence of void nodes by controlling the range of flooding [27]. However, they still cost too much to handle voids.

Graph-based routing algorithms exploit the properties of planar graphs. Some examples are convex face routing [11], original face routing [12], the Face-2 algorithm [13], Other Face Routing (OFR) and Other Adaptive Face Routing (OAFR) in GOAFR [14], and GOAFR+ [15,28]. Some complete void handling techniques in geographic routing protocols include perimeter routing in Greedy Perimeter Stateless Routing (GPSR) [16], Request-Response (RR) in Beacon-Less Routing (BLR) [17], and bypass in Priority-based Stateless Geo-Routing (PSGR) [18]. Theoretically, it has been shown that a planar-graph-based technique guarantees packet delivery [19], because planar graph traversal ensures the discovery of a path if a topologically valid path exists.

Perimeter routing is the complete void handling technique in GPSR [16]. Perimeter routing consists of a planar traversal algorithm, a distributed planarization algorithm and some other protocol optimizations. In GPSR, a planar sub-graph of the original graph is computed during a preprocessing phase using the Relative Neighborhood Graph (RNG) planarization technique or the Gabriel Graph (GG) planarization technique. When a packet becomes stuck at a void node in greedy forwarding, perimeter routing is enabled and the planar traversal algorithm, similar to Face-2 routing [13], is used to walk the stuck packet around the void. The right hand rule is used to walk around the perimeter. The header of a stuck packet carries information, such as the position of the void node, the position of the last intersection that caused a face change, and the first edge traversed on the current face. Such information helps each node make routing decisions locally.

**Figure 3.**
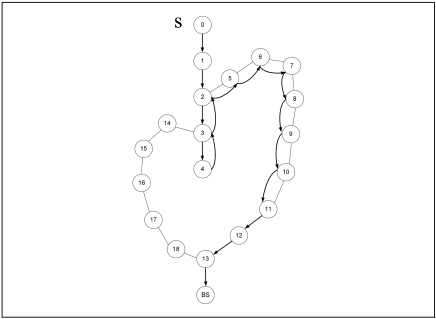
Perimeter mode in GPSR in a closed void.

**Figure 4.**
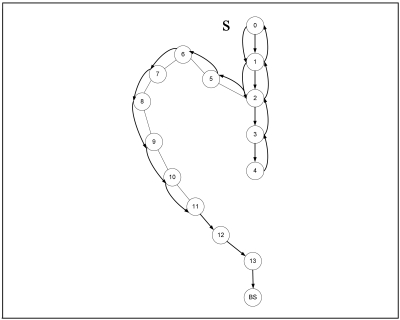
Perimeter mode in GPSR in an open void.

Although GPSR is an accepted stateless location-based routing protocol that guarantees packet delivery if there is a topologically valid path, the detours along the perimeter of its perimeter mode may produce long paths. Planarization in GPSR requires more computational complexity. In GPSR, nodes on the face of the holes may overcrowd due to traffic concentration that may lead to drastic throughput degradation. Excessive energy consumption of void boundary nodes may enlarge the void. Further, GPSR works well in the closed void, but it cannot perform well in an open void. Closed and open are two types of communication void. The closed void is surrounded by sensors, whereas there is an open space to one side in the open void. Figures 3 and 4 illustrate the perimeter mode routing path followed by GPSR in the closed void and open void cases. As shown in Figure 3, a packet travels up to the stuck node (node 4) using greedy forwarding and switches to perimeter mode and travels along the destination using the right hand rule. When it reaches a node that is closer to the destination (i.e. node 11) than the stuck node, it switches back to greedy forwarding. Straight arrows indicate greedy forwarding and curved arrows indicate perimeter mode routing. The open void case is illustrated in Figure 4. After the packet encounters a void, GPSR switches to perimeter mode (node 4) and travels back to the source node. It continues perimeter routing until it reaches node 11 that is closer to the destination than the stuck node (node 4). Once back at node 11, it switches to greedy forwarding.

The right hand rule is inefficiently aggregates data from the sensor to BS. Our algorithm avoids voids without the use of the right-hand rule.

The Distance Upgrading Algorithm (DUA) [20] is a void handling technique that exploits a cost-based idea to handle voids. Similar to the Partial-partition Avoiding Geographic Routing–Mobile (PAGER-M) [21], a packet flows from a node with a higher cost to one with a lower cost. To avoid voids, DUA virtually increases the distance of stuck nodes to the BS such that it can avoid communication voids beforehand.

A cost, which may be equal to its Euclidean distance to the destination, is assigned to each node in the network. The stuck node increases its cost to a value greater than its Euclidian distance to the destination, so that the packet can finally be directed by the high-cost-to-low-cost rule [22] along efficient paths to evade the void.

**Figure 5.**
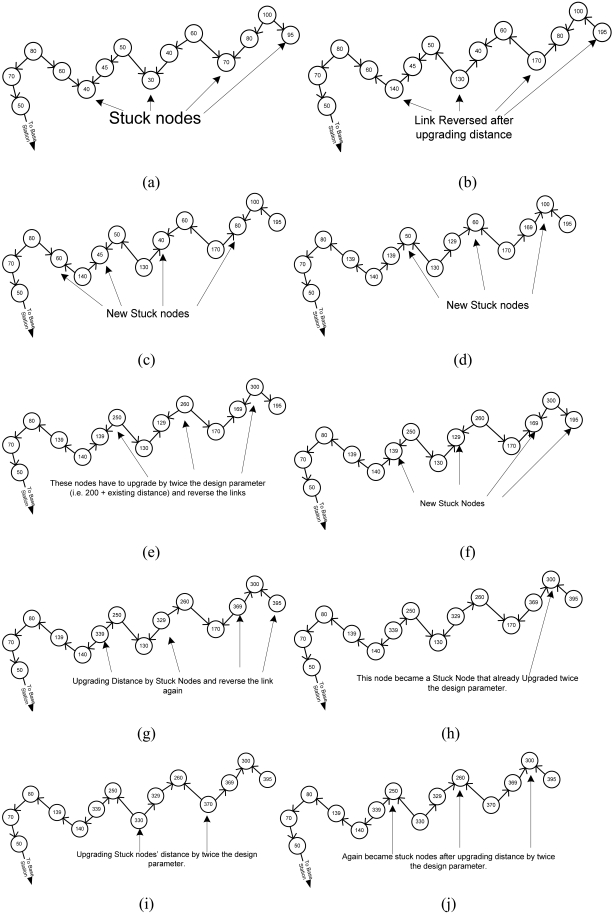
DUA fails in some scenarios.

DUA proves that, after upgrading the distance, all the routing link directions reverse towards BS. The design parameter defined in DUA is greater than the cost of the farthest node from BS. If the source node upgrades distance by twice the design parameter, it reverses all the links. However, this is true if only one or two concave type stuck nodes are considered. If there are more than two concave type stuck nodes, DUA cannot perform well. A concave node is defined in Section 3. Figure 5 illustrates where DUA breaks down. In Figures 5(h) and 5(j), nodes become a stuck node after upgrading the distance by twice the constant.

Distance upgrading by the design parameter in DUA upgrades and downgrades the distance for all nodes along the route from the stuck node to the source node whenever any node becomes a stuck node. This is inefficient for small and many other topological voids. Further, it may select an inefficient route after upgrading the distance, because it increases the cost extensively, such that the packet has to piggyback the location information. Distance downgrading is also required to adjust the distance. This increases the routing overheads in two ways. First, packets carry the node information where it upgraded the distance. Second, BS has to broadcast the control message to downgrade the distance.

It is intuitive that cost-based techniques guarantee packet delivery in connected graphs. Inspired from that, we propose the Void Avoidance Algorithm (VAA) that is better than DUA. In VAA, the stuck node only upgrades the distance and downgrading is not required. There is no need to piggyback the coordinate where the virtual distance upgrade is initialized. The primary focus of our proposed algorithm is to virtually increase the distance to the BS by stuck nodes to turn themselves into non-stuck nodes, to eliminate communication voids. Finally, it forwards packets from sensors to BS along efficient routes using greedy routing. After implementing VAA, no node remains a stuck node in the network, if there is at least one route to the destination. Thus, it is completely distributed, immediately responds to node failure or topology changes, does not require planarizing the network, and incurs substantially less overhead.

## 3. Void Avoidance Algorithm (VAA)

We propose VAA to design a void-free topology for position-based WSNs. The basic idea in our algorithm is to remove all stuck nodes by transforming the routing graph and make a void-free topology. Our algorithm constructs a sink tree from the sensors to BS. Initially a BS broadcasts a hello message, and the reverse broadcast tree is used by the sensors to route packets to the BS. Periodic broadcast of hello message is necessary to maintain the sink tree, because wireless links are dynamic in nature and appear/disappear all the time.

Considering that a packet has to detour around a void from a sensor to a BS, the effective distance between the sensor and the BS is greater than the Euclidian distance. By appropriately upgrading the distances of some sensors, we can direct the packets along the efficient routes towards the BS. We design this algorithm considering real world scenarios, such that if there is no route available to progress to the destination, the algorithm looks behind for a better path and detours intelligently. We handle it creating some virtually ordered distance factors as a cost.

### 3.1. Assumption

We assume that a number of sensor nodes are randomly deployed on an unobstructed roughly plane sensing field with a BS. Sensor nodes participating in the network are aware of the coordinates of their geolocation either from a GPS device or from other means [3-5]. As shown in Figure 2, all sensor nodes within communication range r of a node n are considered as neighbors of n and have bidirectional links with node n. The IEEE 802.11 MAC [23] sends link-level acknowledgements for all unicast packets. Neighbors can communicate directly with each other. The entire sensor nodes know their respective neighbors' geolocation via neighbor discovery protocol. All sensor nodes are static in location. Data packets are sent from sensor to BS. In VAA, each sensor maintains the cost and forwards the packet according to the high-cost-to-low-cost rule. Cost depicts distance from the BS to the sensor, so we call that cost is distance cost (DC).

### 3.2. Distance Cost (DC)

DC from each sensor node to the BS is defined as (TD_k_, …, …, TD_2_, TD_1_, VD). VD is virtual distance, initially set to Euclidian distance (ED) from the node to the BS. TD_1_ … TD_k_ are tag distances and initially set to NULL. These tag distances help void nodes turn into non-void nodes, if the nodes remain void after upgrading VD.

**Figure 6.**
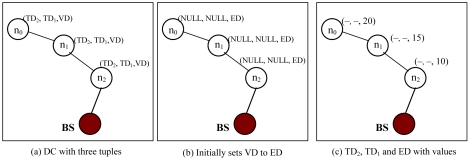
Distance cost.

A study that characterized the impact of routing holes on geographic routing shows that the majority of voids can be circumvented in four hops or less [24]. Considering this fact from [24], we design DC as a three-tuple (TD_2_, TD_1_, VD) that can construct void-free topology. In most cases, two TD fields are sufficient to create void-free topology. Our simulation results in random topology with various average node degree, as in Figure 23, shows the number of upgrades required to circumvent voids. However, it can be increased based on network size. Figure 6 illustrates the DC. Figure 6 (a) shows the TDs and VD's positions. In Figure 6 (b) node n_0_, n_1_ and n_2_ have NULL value for TD_1_ and TD_2_ and ED value for VD. Figure 6 (c) shows the DC with values.

The precedence of DC is in lexicographic order. Our algorithm upgrades stuck nodes' ED temporally that is called VD. It restores the ED if any node finds itself as a non-stuck node.

### 3.3. Six Basic Functions of VAA

Our algorithm performs six basic functions:

- Sends hello message from the BS to advertise its geolocation
- Sends and receives neighbors' information (neighbor discovery protocol)
- Virtual distance upgrading algorithm
- Tag-distance upgrading algorithm
- Finally, after upgrading distance, any sensor can forward a packet using greedy forwarding as per the DC.
- If any new node appears in the void area and makes possible to communicate without virtual distance, then it allows to redirect in the original state and communicate using greedy forwarding.

### 3.4. Algorithm Description

Let F(n) be the set of neighbors of node n. When node n is initialized, it exchanges its location information with its neighbors F(n) using neighbor discovery protocol. It also receives the hello message from the BS and calculates the ED. After receiving the information of neighbors, node n compares DC among neighbors F(n) and then sets logical directional links to the next hop node that is closer to the BS than itself.

**Figure 7.**
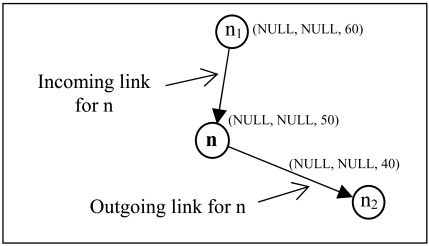
Logical directions.

As shown in Figure 7, node n has an incoming link from node n_1_ and an outgoing link to node n_2_ according to the DC. If DC(n_i_) < DC(n), it is said to be an outgoing link, otherwise it is an incoming link. A logical link (n_i_, n) means n_i_ ∈ F(n). The constructed routing graph is acyclic, because all routing graphs made by the logical links are acyclic [20].

**Figure 8.**
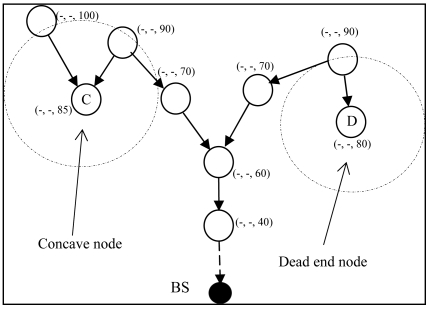
Concave node and dead end node.

If a node does not have an outgoing link, that node is a stuck node. As shown in Figure 8, node C and D are stuck nodes. Let L(n) be neighbor nodes of node n, closer to the BS than node n. Node n is a stuck node if L(n) = ∅. L(n) can be defined as:

(1) $$\text{L}\left(\text{n}\right)=\left\{{\text{n}}_{\text{i}}|\text{DC}\;\left({\text{n}}_{\text{i}}\right)<\text{DC}\;\left(\text{n}\right),\text{for all}\;{\text{n}}_{\text{i}}\in \text{F}\left(\text{n}\right)\right\}.$$

A node is a non-stuck node if L(n) ≠ ∅. Each stuck node falls under one of the following categories:

#### 3.4.1. Concave Node

A node having more than one neighbor, but no one is closer to the BS than itself. Node C in Figure 8 is a concave node. Concave node V(n) can be defined as:

(2) V(n)={n∣F(n)>1,L(n)=∅}.

#### 3.4.2. Dead End Node

A node having no more than one neighbor node and the neighbor is not closer to the destination than it is. Node D in the Figure 8 is a dead end node. Dead end node D(n) can be defined as:

(3) D(n)={n∣|F(n)|=1,L(n)=∅}.

### 3.5. Virtual Distance Upgrading Algorithm

**Figure 9.**
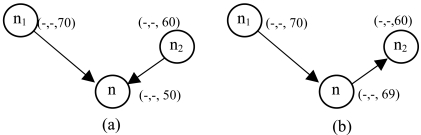
Upgrading virtual distance by concave node.

When node n becomes a concave node, it checks the neighbor table and compares its DC lexicographically. If no neighbor has initialized TD value yet (i.e. TD_1_ = NULL and TD_2_ = NULL) then it selects the highest VD/ED of neighbor and increases its VD just under that of the maximum VD of the neighbor. In Figure 9 (a), node n is a concave node. Neighbor nodes n_1_ and n_2_ have not initialized their TD yet. So, node n upgrades its VD by 69 i.e. maximum VD among neighbors F(n) – 1 (can be written as MaxVD(F(n))-1). After upgrading VD, node n has a L(n) (i.e. n_2_∈L(n)) so it sets the outgoing link to node n_2_. Now, node n is not concave node.

### 3.6. Tag Distance Upgrading Algorithm

If the node is still a concave node after upgrading its VD and/or cannot upgrade its VD further (the condition of VD(n) = MaxVD(F(n)) -1), it upgrades its TD_1_ to MaxTD_1_(F(n))-1, i.e it chooses the value just under that of maximum TD_1_ of neighbors. If no neighbor has a TD_1_ value greater than NULL, it changes its TD_1_ value from NULL to 0 and reverses the link (remember that NULL is smaller than any numerical value in our algorithm).

**Figure 10.**
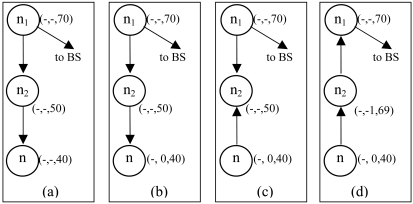
Virtual and tag distance upgrading by dead end node.

Figure 10 illustrates the TD upgrading methods. In Figure 10(a), node n is a D(n) node so TD_1_ sets its value from NULL to 0. Node n_2_ is a V(n) in Figure 10 (c), so it upgrades its VD to just under that of node n_1_'s VD, i.e. 69. After upgrading VD from 50 to 69, node n_2_ is still V(n), so it upgrades its TD_1_ as the maximum TD_1_ of neighbors TD_1_ – 1; i.e. TD_1_ of node n -1 and reverses the link direction. Finally, node n and n_2_ are no longer stuck nodes.

**Figure 11.**
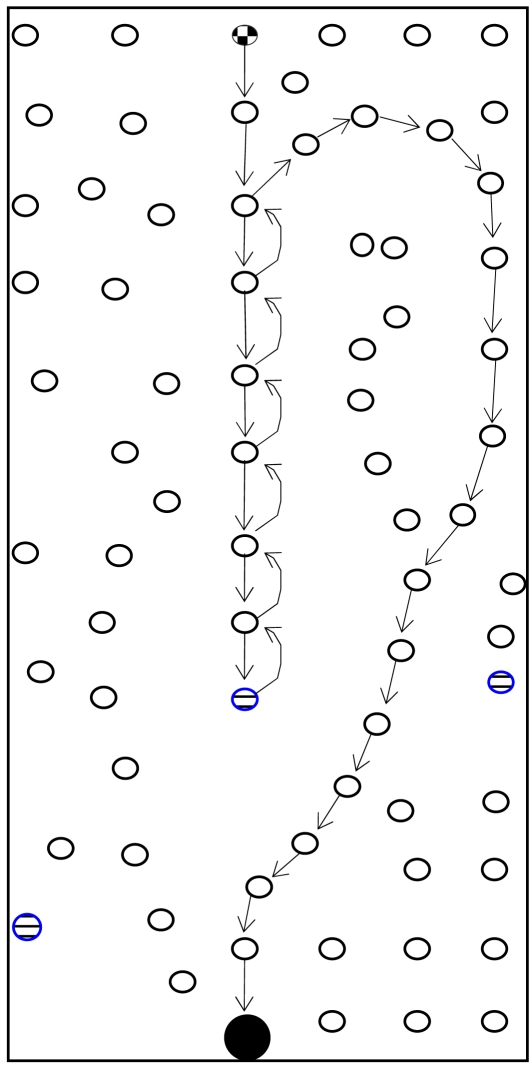
Route by GPSR.

**Figure 12.**
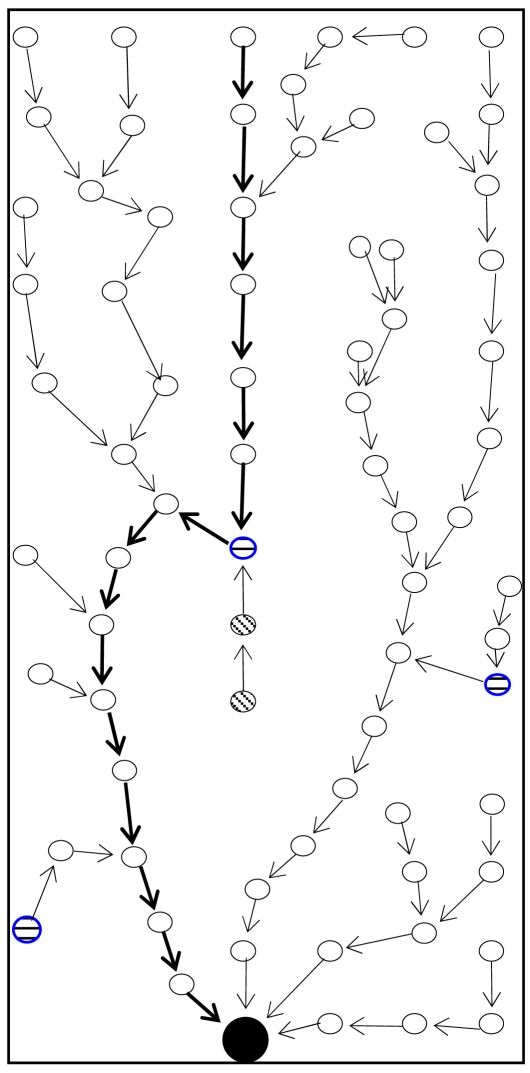
Route after implementing VAA.

If the node is still a stuck node after upgrading its TD_1_, it upgrades its TD_2_ to MaxTD_2_(F(n))-1, i.e. it chooses the value just under that of the maximum TD_2_ of neighbors. If no neighbor has a TD_2_ value greater than NULL, it changes its TD_2_ value from NULL to 0 and reverses the link. Whenever a node n finds itself as a dead end node, it changes its TD value from NULL to 0, reverses links and sends update information to neighbors. As shown in Figure 11, GPSR starts perimeter routing after encountering a stuck node and follows the long route towards BS using the right hand rule. VAA replaces the right hand rule and forms the network topology shown in Figure 12.

### 3.7. Algorithm Implementation

We implement VAA subdividing it into two major subroutines. These algorithm subdivisions run at each sensor node. They are Wake_Up() and Receive_Distance_ Cost_of_Neighbor(). Wake_Up() algorithm executes after node boots up or when the set of neighbors changes. Receive_ Distance_Cost_of_Neighbor() is invoked when any node receives DC from the neighbor nodes. The distributed computation terminates when there is no more notification message. Message loss may mean some dead ends are not removed. This can be handled by neighbors periodically exchanging their distances and by an unremoved dead end executing algorithms to restart the process.

**Figure 13.**
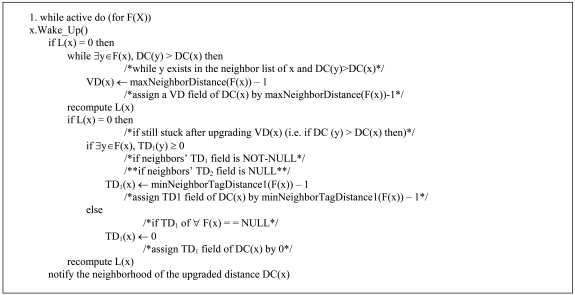

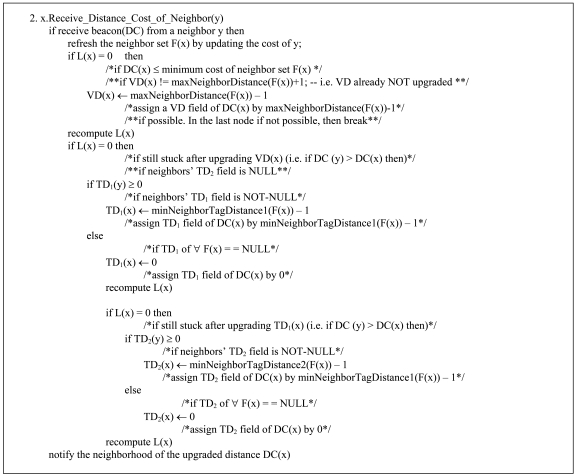
VAA Pseudocode.

**Figure 14.**
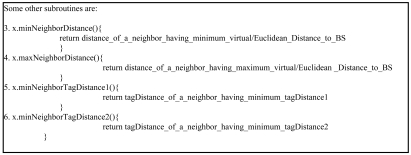
Other subroutines used in VAA.

Table 1 states the notation used in the pseudocode of Figure 13 and Figure 14. Figure 13 is the pseudocode for VAA and Figure 14 that of some other main subroutines used in VAA.

Other subroutines support the main subroutines. minNeighborDistance() calculates the distance of a neighbor having minimum VD/ED to BS. maxNeighborDistance() computes distance of a neighbor having maximum VD/ED to BS. minNeighborTagDistance() computes TD of a neighbor having minimum TD.

## 4. Evaluation

This chapter shows the result of simulating different scenarios to evaluate VAA performance. We simulate the algorithm on a variety of static wireless sensor network environments. We simulate GPSR and DUA to compare the performance of VAA to previous work in wireless routing. We selected those two routing protocols since GPSR is an accepted stateless location-based routing protocol and our work is closer to DUA in that it replaces the existing right hand rule to avoid communication voids.

### 4.1 Simulation Environment

In the present work, we use the event driven simulator ns-2 [25] for our simulations. The network consists of 100 nodes for predefined confined in a 500 × 600 m^2^ area. Transmission range of each node is assumed 40 m. The simulation runs for 500 seconds. BS is located near the middle-left at (0, 300). We simulate three CBR flows originating from randomly chosen nodes across the network. Each flow sends 32 byte packets at 256 bps.

We generate and evaluate different possible random and predefined scenarios for simulation. The key performance measures are path length, energy consumption and routing overhead. The results presented here are the average values taken from multiple simulation results. We use Gabriel Graph (GG) for our simulation with beacon interval of 5 seconds. The other simulation parameters are given in Table 2.

We draw a virtual line from source to destination and divide the area into two sides, namely L side and R side. Different possible scenarios are generated and visualized using Network Animator (NAM) [26].

### 4.2. Experimental Results

When there is no void, VAA, GPSR and DUA have similar path length, because all of them carry out greedy routing in a void-free scenario.

**Figure 15.**
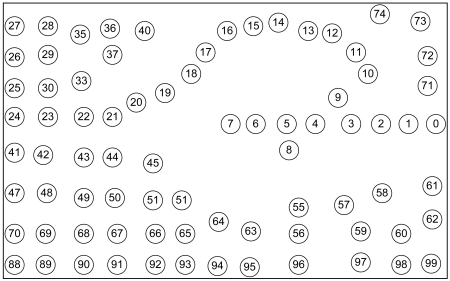
100 node topology with one communication void.

VAA outperforms GPSR significantly in cases with an open void. GPSR follows an inefficient route if there is an open void on the R side as shown in Figure 15. The packet from the source node 0 destined to the BS (node 24) travels upto node 7 (i.e., n_0_ → n_7_, where “→” indicates the sequence of nodes strictly monotonically increasing from n_i_, n_i+1_, …, …, n_i+n_) according to the greedy rule. Greedy forwarding fails in node 7, because node 7 is a void node. After that, it forwards packets according to the perimeter rule to the next face towards node 8 using the right hand rule. Then the packet travels on the respective face of n_7_-n_6_-n_5_-n_8_-n_5_-n_4_-n_3_-n_2_-n_1_-n_0_-n_1_-n_2_-n_3_-n_9_→n_24_ nodes.

**Figure 16.**
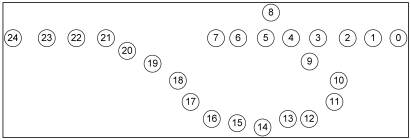
Open void on L side.

In Figure 16, the open void is in the L side. It is the reverse topology case of Figure 15 (in Figure 15, open void is on the R side). In this topology, GPSR travels n_0_→n_7_-n_6_-n_5_-n_4_-n_3_-n_9_→n_24_. This is the best route GPSR can travel, however VAA travels n_0_-n_1_-n_2_-n_3_-n_9_→n_24_ 19 hops, still less than the route traveled by GPSR, 27 hops, since GPSR switches to recovery mode after encountering the communication void. However, VAA prevents entering the void and it detours from node 3 toward node 9 in Figure 16.

**Figure 17.**
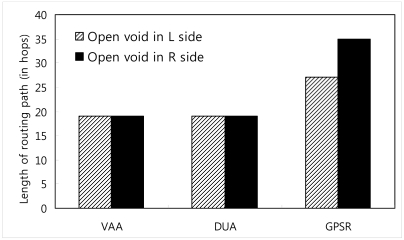
Length of routing path when there is one open void.

The graph in Figure 17 shows GPSR travels the longest path in the open void in R side case (scenario in Figure 15), more than in any other case. However, VAA and DUA travel a similar path (i.e. n_0_→n_3_-n_9_ →n_24_) in either case (i.e. open void in L side and R side) but GPSR travels the longer path.

**Figure 18.**
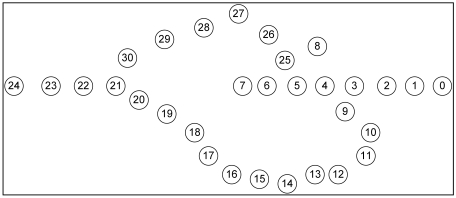
Closed void.

In the case of a closed void, as in Figure 18, GPSR travels the longest route n_0_→n_7_-n_6_-n_5_-n_4_-n_3_-n_9_→n_24_, 27 hops. However, DUA and VAA travel n_0_→n_6_-n_25_→n_30_-n_21_→n_24_, only 16 hops.

**Figure 19.**
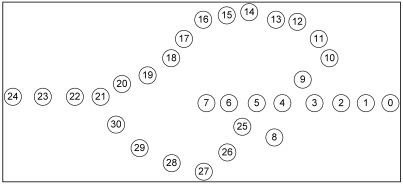
Closed void (best case scenario).

Even in a best case scenario (i.e. flipping Figure 18 horizontally) in Figure 19, all three protocols take a similar path. Due to the nature of GPSR, packets go inside the void to node 7, and then come back thus n_0_→n_7_-n_6_-n_25_→n_30_-n_21_→n_24_. DUA and VAA do not go inside the void so they detour from node 6, so the route is n_0_→n_6_-n_25_→n_30_-n_21_→n_24_. This is still fewer hops than GPSR.

**Figure 20.**
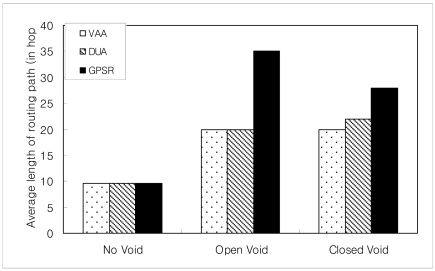
Average length of routing path in different types of void.

The average length of the routing path in VAA is similar in both cases of closed and open void. DUA may select an inefficient routing path, due to its nature, to upgrade virtual distance too high as described in section 2 (for details refer [20]). So, as shown in Figure 20, in the case of closed void, DUA follows a longer route than VAA. VAA travels the best route in either case.

**Figure 21.**
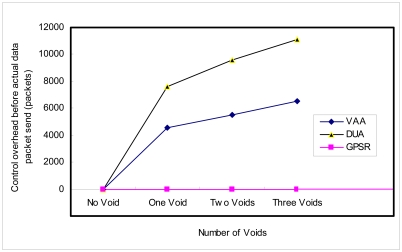
Control overhead (generated by routing) before sending data packets.

VAA exchanges neighbor information via the Hello message of the neighbor discovery protocol including distance update information. Thus, there is no additional control overhead. Hello messages broadcast once in every five seconds. However, before sending the actual data packet, all nodes assure their next hop node to BS. For this, VAA takes less time than DUA. DUA takes significantly more time when there is more than one void. DUA also takes more time, because it upgrades virtual distance up to the source from the void node. Figure 21 shows control packet overhead before sending the actual data packets. The graph shows VAA has 40% less overhead than DUA where a void exists. There is no prior setup in GPSR so it does not have control packet overhead, before sending actual data packet, except for the neighbor discovery protocol.

**Figure 22.**
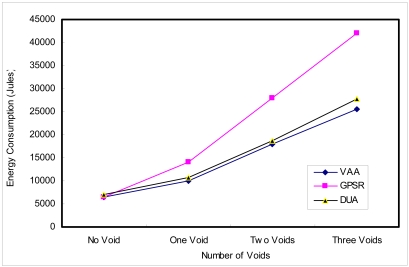
Energy consumption.

GPSR's energy expenditure ratio is higher, because it follows a longer routing path than VAA. DUA follows a similar path, but in some cases of the closed void, it follows a longer path. DUA has a more control overhead (generated by routing) and in case of the topology changes, all nodes up to the destination have to change the virtual distance. Thus, more energy is consumed in DUA than VAA. Figure 22 shows the energy expenditure in different cases. Where voids exist, energy consumption is 28% - 39% more in GPSR than VAA. However, DUA consumes 8% more energy where there are three voids. The ratio increases as the number of voids increases. Energy efficiency of VAA is due to its low control overhead and low path length, as shown previously.

**Figure 23.**
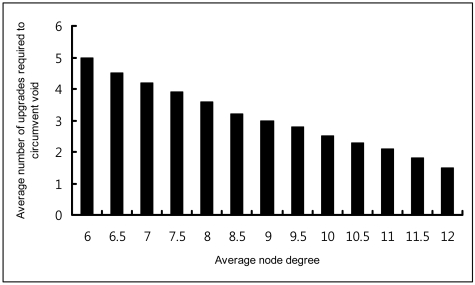
Average number of upgrades required to circumvent voids with different average node degree.

Figure 23 shows the simulation in random scenarios for the average number of distance upgrade required to circumvent the voids. These outcomes are the average of 50 simulation results of the network consists of 350 nodes to 700 nodes confined in a 500 × 600 m^2^ area. In denser scenarios, when average node degree ((πr^2^n)/A, where, n is the number of nodes within a node n_i_'s transmission range r and A is a total coverage area) is high, only a few upgrades are required. On the other hand, when the average node degree decreases there are more chances to be dead end nodes and concave nodes and it also requires more upgrades in virtual distance to circumvent voids. However, in any of the cases majority of the voids can be avoided within four upgrades or less. In average cases it requires only around three upgrades to circumvent.

## 5. Conclusions and Future Work

A new void avoidance algorithm for geo-routing that forms a void-free wireless sensor network is presented in this paper. Simulation results show that VAA can evade the communication voids with small routing overheads. VAA increases the distance and decides the next hop node via a cost function that has three tuples to give efficient resolution for the dead end or concave node. Energy consumption is low and it selects an efficient communication path.

Our study is limited to static wireless position-based sensor networks. In the future, it can be extended to limited mobile or highly mobile wireless position-based sensor networks. Moreover, we simulated this algorithm only for a single BS. It can be extended to multiple BSs. We also left unresolved some important areas, such as efficient positioning, maximum utilization of resources, energy conservation and localization for the future research purpose.

## Figures and Tables

**Table 1.** Notations.

**F(X)**	Entire set of nodes
**L(x)**	Neighbor of node x closer to the BS than x
**F(x)**	Neighbors of node x
**VD(x)**	Virtual distance of node x initially set to the Euclidian distance to BS
**TD_1_(x)/TD_2_(x)**	Tag distances of node x, initially set to NULL
**DC(x)**	Distance cost of node x. i.e. (TD_2_, TD_1_, VD/ED)
**←**	Assignment operator
**ED(x)**	Euclidean distance from node x to BS

**Table 2.** Simulation parameters.

Simulator	NS-2 version 2.29
Simulation area	500 × 600m^2^
Number of nodes	100 (predefined scenario)
Initial energy	1,000 J
Transmitting energy	0.06 J
Receiving energy	0.042 J
Idle power	0.02 w
Transmission radio range	40 m
Connection type	UDP
Duration	500 seconds
Agent and application	CBR over UDP
MAC	802.11
Link bandwidth	2 Mbps
Antenna	Omni Antenna
Interface queue	DropTail/PriQueue
Mobility	[0]m/s (static)
Traffic model	CBR over UDP
Data packet size	32 byte
Bacon interval	5 seconds
